# A decade of intestinal protozoan epidemiology among settled immigrants in Qatar

**DOI:** 10.1186/s12879-016-1728-3

**Published:** 2016-08-05

**Authors:** Marawan A. Abu-Madi, Jerzy M. Behnke, Sonia Boughattas, Asma Al-Thani, Sanjay H. Doiphode

**Affiliations:** 1 Department of Biomedical Science, Biomedical Research Centre, College of Health Sciences, Qatar University, P.O. Box 2713, Doha, Qatar; 2 School of Life Sciences, University of Nottingham, University Park, Nottingham, NG7 2RD UK; 3 Department of Laboratory Medicine and Pathology, Hamad Medical Corporation, P.O. Box 3050, Doha, Qatar

**Keywords:** Protozoa, *Blastocystis hominis*, *Giardia duodenalis*, *Entamoeba*, Non-pathogenic amoebae, Qatar, Immigrants, Long-term residents

## Abstract

**Background:**

The World Health Organization estimates that about 3.5 billion people worldwide are affected by intestinal parasitic infections. Reports have already emphasized the role of immigrants in outbreaks of parasitic diseases in industrialized countries. With the mass influx of immigrants to Qatar, patent intestinal parasitic infections have been observed. Herein, the prevalence of intestinal protozoan infections was analysed in 29,286 records of subjects referred for stool examination at the Hamad Medical Corporation over the course of a decade (2005 to 2014, inclusive).

**Results:**

Overall prevalence of combined protozoan infections was 5.93 % but there were significant temporal trends, age and sex effects and those arising from the region of origin of the subjects. The most common protozoan was *Blastocystis hominis* (overall prevalence 3.45 %). *Giardia duodenalis*, *Chilomastix mesnili, Entamoeba coli, Entamoeba hartmanni, Endolimax nana, Iodamoeba butschlii, Entamoeba histolytica/dispar, Cryptosporidium* sp. and a single case of *Isospora* were also detected. The prevalence of combined protozoan infections, *G. duodenalis* and the non-pathogenic amoebae all declined significantly across the decade. That of *B. hominis* varied between years but showed no directional trend across years and there was no evidence that prevalence of *E. histolyitica/dispar* changed significantly. Protozoan infections were observed among all regional groups, but prevalence was higher among subjects from the Arabian Peninsula, Africa and Asia compared to those from the Eastern Mediterranean and Qatar. Prevalence was higher among male subjects in all cases, but age-prevalence profiles differed between the taxa.

**Conclusion:**

These results offer optimism that prevalence will continue to decline in the years ahead.

## Background

Currently, it is estimated that 160 million international migrants, mostly originating from low/middle income countries live in high-income countries and this figure could rise to 405 million by the year 2050 [1]. The reasons for human migration are complex and in most cases include a variety of social, political and economic factors, as illustrated by the huge numbers of migrants arriving recently in the European Union from Syria, Afghanistan, Iraq and other countries hit by regional poverty, instability and conflict [2]. Some reports have already emphasized the role of immigrants in outbreaks of parasitic diseases in Europe and America, with enteric infections being among the most frequently encountered [3, 4].

The World Health Organization estimates that about 3.5 billion people worldwide are affected by intestinal parasitic infections and that 450 million developed clinical illness [5]. These intestinal parasitic infections are more prevalent in developing countries (30 to 60 %) than in developed ones (≤2 %) and consequently often receive less attention from public health authorities in the latter [6]. Most intestinal protozoan infections, which are largely dependent on the faecal-oral route of transmission, are unlikely to be transmitted efficiently in the context of Western societies and developed countries where clean water is readily available and reliable public sanitation is common. However, protozoa including *Giardia duodenalis* and *Entamoeba histolytica* are frequently reported, associated with sporadic outbreaks of disease in industrialized countries and have been attributed in part to mobile populations [7, 8]. Both parasites are responsible for a significant morbidity and mortality worldwide and their transmission is highly dependent on the faecal-oral route via contaminated foods and water sources [9].

With the mass influx of immigrants to Qatar, encouraged by its rapid socio-economic development, patent intestinal parasitic infections have been observed and monitored [10]. In earlier analyses the prevalence of intestinal protozoa was reported to be 7.98 % over the period 2005–2008 [11] but falling trends in prevalence were observed in the next 3 years (2009–2011) with prevalence declining to 5.13 % for all the protozoa combined [12]. Here we continue analysing the data over a period of a further 3 years (2012–2014) to ascertain whether the declining trends have been sustained in the longer-term. The current paper therefore focuses on temporal trends in a dataset that spans an entire decade (2005–2014).

## Methods

### Study subjects and sample collection

This study was based on a retrospective survey of intestinal parasitic infections based on the records held at Hamad Medical Corporation (HMC) data-base (MediCom) maintained at the Department of Laboratory Medicine and Pathology at HMC and its outpatient clinics between 2005 and 2014. We examined 33,665 records of patients referred to different departments of the HMC hospitals including maternity, paediatrics, internal medicine and gastroenterology, and who participated in a routine stool test.

We combined two previously analysed dataset for the period from 2005 to 2008, and 2009 to 2011 with that for the following 3 years to December 31^st^, 2014. We removed 1951 records of children from 2 days to less than 7 months of age in order not to bias long-term trends in our analysis, since the earlier analysis from 2005 to 2008 had been confined to those over 7 months of age. Among this group, there was only one record of infection and this was with *Endolimax nana* in a 3-month-old Qatari girl in 2010.

A further 706 records comprising subjects from Europe (*n* = 338), North America (*n* = 310), Central America (*n* = 11), Australasia (*n* = 25), South America (*n* = 19) were also removed, because these continents had not been considered in our earlier analyses. Three more records were removed because of unknown nationality of the subjects. Among these 706 subjects, 29 were infected with various combinations of protozoan parasites (prevalence = 4.1 %, CL_95_ = 2.88–5.82). There were 23 cases of *Blastocystis hominis,* two of *G. duodenalis,* two of *Entamoeba coli*, four *E. nana* and one of *Iodamoeba butschlii* among this group, with three subjects infected concurrently with two species (*B. hominis* + *E. nana*, *B. hominis* + *E. coli* and *E. nana* + *I. butschlii*).

### Stool examination

Stool samples were obtained from subjects referred for examination at HMC as part of a routine screening policy for the diagnosis of diseases associated with intestinal infections. Confidentiality was maintained throughout and the identity of subjects was not available to us, other than through each individual’s reference number. Age, sex and geographical region were recorded for each patient prior to taking the specimen. Fresh stool specimens were collected in 25 ml clean wide-mouth, covered plastic containers. Stool samples were then immediately transported to the Microbiology Laboratory at HMC [13].

Stool examination was carried out in a safety cabinet, where stool specimen was preserved in an ecofix preservative vial (Meridin Biosciences, Inc.). The contents were stirred with fine clean disposable wooden sticks to remove large clumps and mixed vigorously by vortex to homogenize the sample. To ensure adequate fixation of the homogenized stool, the sample was kept for half an hour at room temperature. The preserved specimen was mixed by vortex and filtered through a macro-con filtration unit for the removal of bulky debris. After filtration, 10 % formalin and ethyl acetate were added, the sample was centrifuged for 10 min at 3000 rpm and the fluid containing diethyl ether and formalin was discarded. The pellet was re-suspended by agitation, poured onto a microscope slide containing one drop of iodine and examined microscopically for the presence/absence of parasite eggs/cysts and to enable identification of parasites in positive samples. Amoeba species other than *E. histolytica/dispar* including *E. coli*, *Entamoeba hartmanni*, *E. nana, Chilomastix mesnili* and *I. butschlii* were pooled together and recorded as non-pathogenic amoebae in the first period (2005–2008) because the cysts are not easily distinguishable [11]. However, in the second and third periods, these stages were separated by the microscopists and relevant data are presented. Throughout we refer to *G. duodenalis* rather than *G. lamblia* and *G. intestinalis,*

### Definition of variables

All birth dates and examination dates were recorded meticulously and the ages of subjects were classified into ranges by years. Thirteen age classes were then constructed to span ≤1 year, 1.1–1.9, 2.0–4.9, 5.0–9.9, 10.0–14.9, 15.0–19.9, 20.0–29.9, 30.0–39.9, 40.0–49.9, 50.0–59.9, 60.0–69.9, 70.0–79.9 and < 79.9 years.

The subjects in this study came from 69 countries. For the purpose of analysis, the subjects were grouped into four geographical groups for comparison with Qatari nationals (*n* = 9357). These were as follows: from six countries in the Arabian Peninsula (*n* = 1441, Kuwait, Bahrain, Oman, Saudi Arabia, United Arab Emirates and Yemen); from seven countries in the Eastern Mediterranean (*n* = 2799, Iraq, Jordan, Lebanon, Palestine, Syria and Turkey); from 31 countries in Africa (*n* = 5354, Algeria, Benin, Burkina Faso, Cameroon, Chad, Comoros, Djibouti, Egypt, Eritrea, Ethiopia, Gambia, Ghana, Guinea, Kenya, Liberia, Libya, Malawi, Mali, Mauritania, Mauritius, Morocco, Mozambique, Niger, Nigeria, Senegal, Somalia, South Africa, Sudan, Tanzania, Tunisia and Uganda); and from 25 countries in Asia (*n* = 10,335, Afghanistan, Azerbaijan, Bangladesh, Bhutan, Burma, China, India, Indonesia, Iran, Japan, North Korea, South Korea, Kyrgystan, Malaysia, Maldives, Nepal, Pakistan, Philippines, Singapore, Sri Lanka, Tajikistan, Thailand, Turkmenistan, Uzbekistan and Vietnam). Note that unlike earlier two papers [11, 12], Kuwait was classified here as part of the Arabian Peninsula.

The analysis was based on data recorded at Department of Laboratory Medicine and Pathology, HMC from the 1^st^ of January 2005 until the 31^st^ of December 2014, and is coded by year of study. However, since an analysis has already been published for the period 2005–2008 [11], and for a comparison of 2005–2008 with 2009–2011 [12], we call these periods 1 and 2 respectively, and in some analyses compare prevalence rates in these periods with period 3 covering the years 20012–2014.

### Statistical analysis

Prevalence data (percentage of subjects infected) are shown with 95 % confidence limits (CL_95_), calculated as described employing bespoke software [14]. Prevalence was analysed by maximum likelihood techniques based on log linear analysis of contingency tables using the software package SPSS (Version 22.0.0). Initially, full factorial models were fitted, incorporating as factors SEX (2 levels, males and females), AGE (13 levels as shown in Table 1), YEAR of study (10 levels, for each of the years from 2005 to 2014) and REGION of origin (5 levels, Africa, Arabian Peninsula, Asia, Eastern Mediterranean and Qatar). In some analyses PERIOD was fitted rather than YEAR because we wanted to know whether prevalence had changed between the Period 1 (2005–2008), Period 2 (2009–2011) and Period 3 (2012–2014). The presence/absence of a parasite or parasites was considered as a binary factor and is referred to as INFECTION in the analysis as described previously [12].

**Table 1:** Prevalence (%) of protozoan parasites in the study population in the first (2005–2008), second (2009–2011), third (2012–2014) periods and overall

	Prevalence (95 % confidence limits)			
	Period 1	Period 2	Period 3	Combined ***
	2005–2008	2009–2011	2012–2014	
Protozoa*				
*Blastocystis hominis*	4.32 (3.907–4.738)	2.78 (2.439–3.124)	3.26 (2.930–3.586)	3.45 (3.240–3.658)
*Giardia duodenalis*	1.94 (1.662–2.226)	1.44 (1.188–1.684)	1.10 (0.911–1.297)	1.47 (1.331–1.606)
*Chilomastix mesnili*	Nd	0.05 (0.012–0.116)	0.03 (0.006–0.078)	0.03 (0.014–0.072)
*Entamoeba coli*	Nd	0.50 (0.361–0.668)	0.45 (0.338–0.597)	0.47 (0.383–0.578)
*Entamoeba hartmanni*	Nd	0.03 (0.007–0.099)	0.02 (0.002–0.064)	0.02 (0.008–0.058)
*Endolimax nana*	Nd	0.88 (0.697–1.101)	0.53 (0.400–0.677)	0.68 (0.568–0.796)
*Iodamoeba buetschlii*	Nd	0.15 (0.078–0.251)	0.13 (0.075–0.220)	0.14 (0.093–0.202)
All Non pathogenic amoebae**	2.52 (2.199–2.840)	1.38 (1.136–1.623)	0.95 (0.773–1.132)	1.57 (1.432–1.717)
Entamoeba histolytica/dispar**	0.29 (0.193–0.427)	0.23 (0.138–0.349)	0.12 (0.068–0.209)	0.21 (0.159–0.268)
*Cryptosporidium* sp.	Nd	0.05 (0.012–0.116)	0.06 (0.025–0.128)	0.05 (0.027–0.098)
All protozoa combined	7.98 (7.429–8.536)	5.13 (4.673–5.593)	4.89 (4.488–5.286)	5.93 (5.664–6.205)

*In addition to the species listd above there was one case of *Isopora* sp. identified in a 57 year-old male Sudanese subject

** Non-pathogenic amoebae are *E. coli*, *E. hartmanni*, *E. nana* and *I. buetschlii*

Pathogenic amoebae *E. histolytica*/*dispar* which cannot be distinguished on cyst morphology. See text for further explanation

*Nd* not done, these species were not assessed independently in Period 1

***Overall prevalence across Periods 1, 2 and 3 combined or Periods 2 and 3 combined when relevant data for Period 1 were not available

## Results

Of the 29,286 subjects who met the inclusion criteria, 1738 (5.93 %, CL_95_ = 5.664–6.205) were infected with protozoan parasites. The overall prevalence of each of the species over the 10-years period of the study is given in Table 1, and prevalence is shown also for the Periods 1, 2 and 3 as delimited in the Methods section. These data show that there was a continuing trend of falling prevalences for most species from Period 1 to Period 3 (*G. duodenalis*, pathogenic and combined non-pathogenic amoebae, and all protozoa combined). Even among the four species of non-pathogenic amoebae, which were only differentiated in Periods 2 and 3, prevalence was lower in Period 3 compared with Period 2. The only exception was *B. hominis* whose prevalence in Period 3 was lower than in Period 1, but higher than in Period 2.

### Temporal changes

**Fig. 1:**
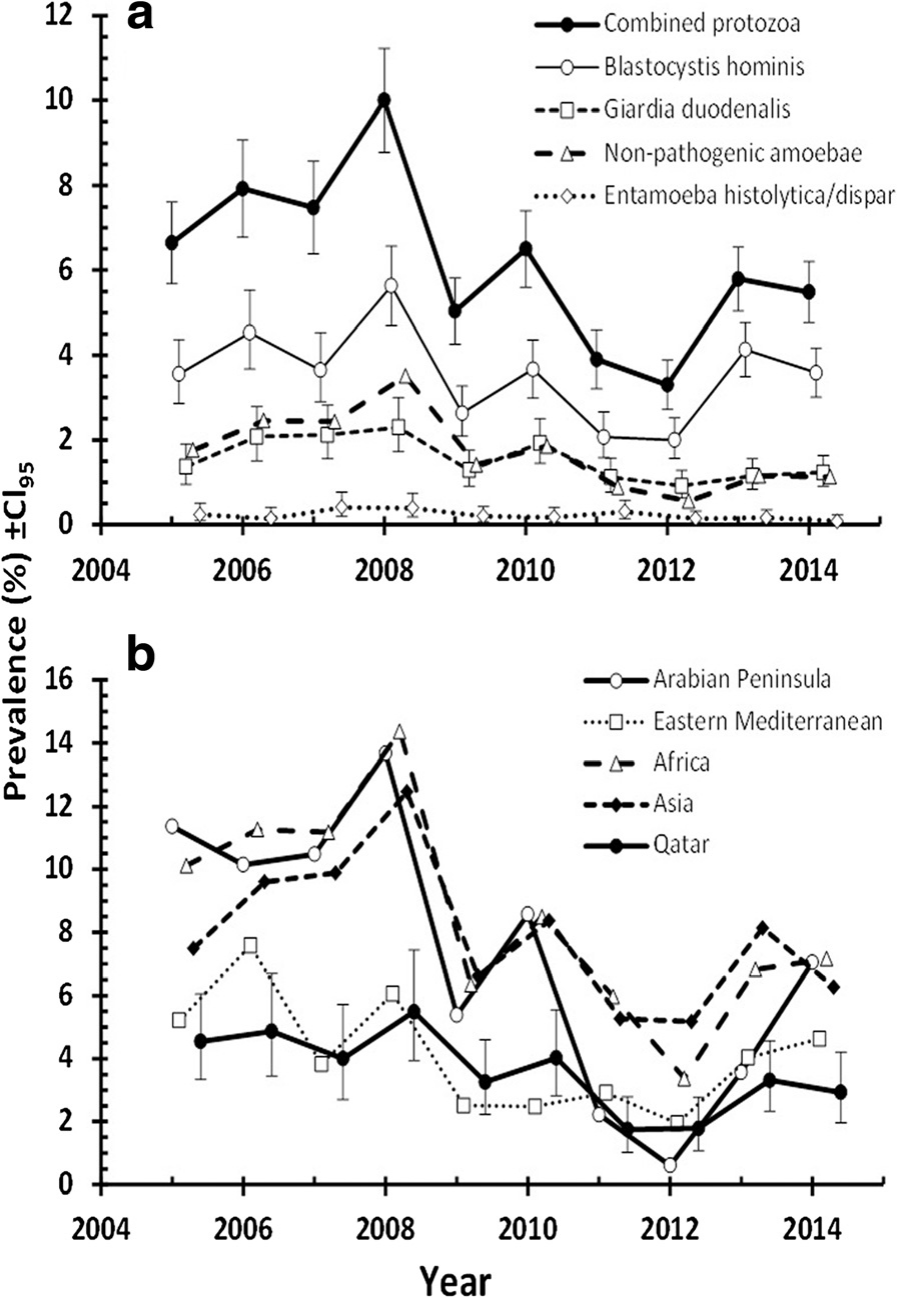
Temporal changes in the prevalence of combined protozoan infections and for specified taxa in the study population (**a**), and of combined protozoan infections among the five regional subsets of the population. Error bars in B, are shown only for the Qatari population so as not to obscure the temporal trends. Sample sizes in A are 29,286 for all taxa and in **b**, Arabian Peninsula = 1441; E. Mediterranean =2799; Africa = 5354; Asia = 10,335 and Qatar =9357

Four taxa (*B. hominis*, *G. duodenalis*, combined non-pathogenic amoebae and *E. histolytica/disapar*) were recorded across the whole of the 10-years period (Table 1). Figure 1a shows prevalence of the combined protozoan infections and of each of these four taxa individually across the whole of the decade. The effect of YEAR was significant for combined protozoa (*χ*^2^_9_ = 167.4, *p* < 0.001), *B. hominis* (*χ*^2^_9_ = 93.4, *p* < 0.001), *G. duodenalis* (*χ*^2^_9_ = 46.7, *p* < 0.001), and combined non-pathogenic amoebae (*χ*^2^_9_ = 115.1, *p* < 0.001), but not for *E. histolytica/dispar*. However, a significant decline in prevalence with increasing year was found only for all protozoa combined (*R*_*s*_ = -0.673, *n* = 10, *p* = 0.033), non-pathogenic amoebae (*R*_*s*_ = -0.721, *n* = 10, *p* = 0.019) and *G. duodenalis* (*R*_*s*_ = -0.679, *n* = 10, *p* = 0.025), but not in the case of *B. hominis* (*R*_*s*_ = -0.261, *n* = 10, *p* = 0.47) or *E. histolytica/dispar* (*R*_*s*_ = -0.552, *n* = 10, *p* = 0.098). Thus, only *E. histolytica/dispar* infections failed to show between–year variation and not surprisingly despite a weak negative trend with successive years, no directional change over time, suggesting a degree of stability. In contrast, the prevalence of *B. hominis* fluctuated significantly between years but overall failed to show a directional change over time.

### Region of origin of subjects

In all four taxa and when combined, there was a highly significant effect of REGION (Table 2; all four taxa combined, *χ*^2^_4_ = 216.6, *p* < 0.001; *B. hominis, χ*^2^_4_ = 67.1, *p* < 0.001; *G. duodenalis, χ*^2^_4_ = 103.4, *p* < 0.001; non-pathogenic amoebae, *χ*^2^_4_ = 105.1, *p* < 0.001; *E. histolytica/dispar, χ*^2^_4_ = 23.2, *p* < 0.001). All four taxa were detected among subjects from each of the five regions, with the lowest prevalence of combined protozoa from among the Qatari and Eastern Mediterranean populations. Those from the Arabian Peninsula, Africa and Asia showed prevalence values that were twice as high and similar among the subjects from these regions (Table 2). *G. duodenalis* and *E. histolytica/dispar* were most common among the Asians, while the highest prevalence values for *B. hominis* and non-pathogenic amoebae were from among the Africans. *E. histolytica/dispar* was particularly rarely encountered among the Qataris and subjects from the Arabian Peninsula, while *G. duodenalis* was rarest among the Qataris and those from the Eastern Mediterranean region.

**Table 2:** No of subjects in each category and the prevalence (%) of the four protozoan taxa and combined protozoa by host sex, and region of origin

	No. Subjects	*B. hominis*	*G. duodenalis*	*Non-pathogenic amoebae*	*E. histolytica/dispar*	Combined protozoa
Host sex						
Males	16991	***4.20***	***1.66***	***1.73***	***0.26***	***6.81***
Females	12295	2.58	1.20	1.36	0.14	4.73
Region						
Arabian Pen.	1441	3.61	1.87	1.94	0.07	6.94
Eastern Med.	2799	2.82	0.54	0.86	0.14	3.97
Africa	5354	***4.33***	1.68	***2.58***	0.28	***7.68***
Asia	10335	4.31	***2.24***	1.96	***0.36***	7.63
Qatar	9357	2.16	0.72	0.73	0.04	3.49

The statistical outputs were derived from minimum sufficient models, after first fitting for each species in turn, all variables into a single full factorial model, and then stepwise backward deletion of non-significant terms

The *χ* ^2^ values for goodness of fit of the minimum sufficient models for *B. hominis*, *G. duodenalis*, non-pathogenic amoebae, *E. histolytica/dispar* and combined protozoan infections were as follows: 1536.2 (df = 1705, *P* = 0.99), 1322.6 (df = 1801, *P* = 1), 1232.5 (df = 1728, *P* = 1), 847.0 (df = 1823, *P* = 1) and 1350.1 (df = 1420, *P* = 0.91), respectively. The importance of each factor in the final minimum sufficient model for each taxon is given in the text. Additional terms in the final models, that did not incorporate the presence/absence of parasites are not shown, but can be made available on request from the authors

### Changes in prevalence by region

Figure 1b shows that the time-course of changes in prevalence of combined protozoan infections varied between subjects from different regions. The sharpest downward trends were detected for subjects from Africa and the Arabian Peninsula both of which were significant (*R*_*s*_ = -0.661, *n* = 10, *p* = 0.038; *R*_*s*_ = -0.733, *n* = 10, *p* = 0.016). The change of prevalence with time for Qatari nationals was modest by comparison because prevalence among this group initially was low, but nevertheless, it was highly significant (*R*_*s*_ = -0.709, *n* = 10, *p* = 0.022). Prevalence among those from the Eastern Mediterranean and Asia did not show a significant decline with time (*R*_*s*_ = -0.442, *n* = 10, *p* = 0.2; *R*_*s*_ = -0.564, *n* = 10, *p* = 0.09, respectively) even though in both cases the correlations were negative implying a downward trend in prevalence with time.

### Age of subjects

**Fig. 2:**
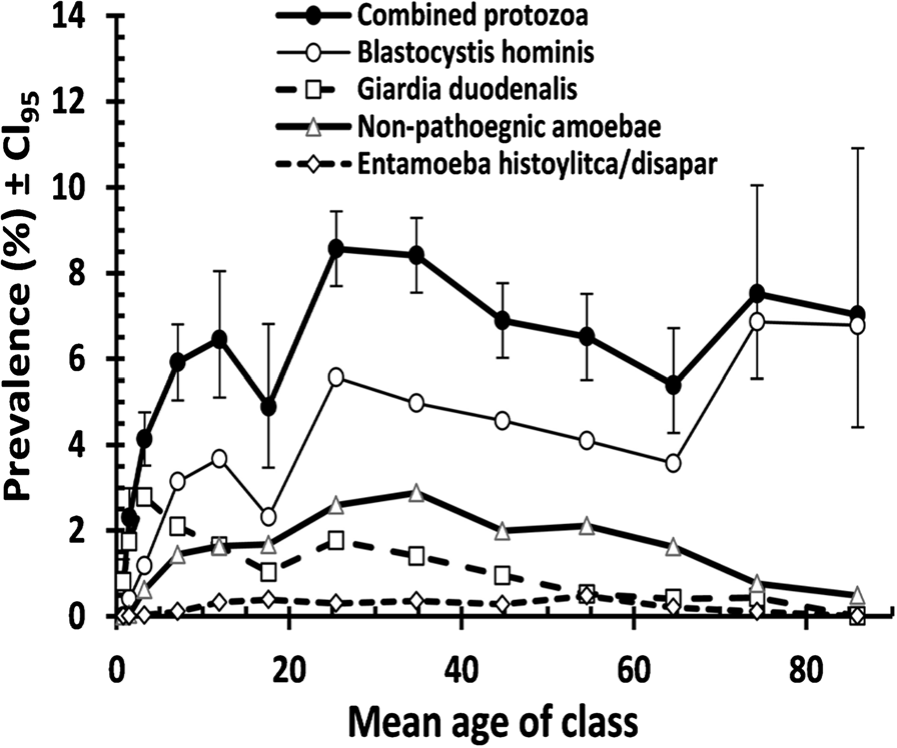
Age-prevalence profile for all four tax and for combined protozoan infections. The sample sizes for age classes 1–13 were *n* = 1867, 2473, 3891, 2769, 1224, 777, 3968, 3921, 3264, 2318, 1483, 918 and 413 respectively

For all four species combined and for each of the individual species, there was a highly significant effect of age (all four taxa combined, *χ*^2^_12_ = 333.2, *p* < 0.001; *B. hominis, χ*^2^_12_ = 445.0, *p* < 0.001; *G. duodenalis, χ*^2^_12_ = 118.9, *p* < 0.001; non-pathogenic amoebae, *χ*^2^_12_ = 171.2, *p* < 0.001; *E. histolytica/dispar, χ*^2^_12_ = 37.1, *p* <0.001). The age prevalence profiles are illustrated in Fig. 2, where the contrasting patterns of these profiles can be seen. For *B. hominis,* peak prevalence was in the oldest age classes (age class 12 with a mean age = 74.3, *n* = 918 and age class 13 with a mean age = 86.0 years, *n* = 413). For *G. duodenalis,* the peak was among the youngest age classes (age class 3, *n* = 3891 with a mean age of 3.2 years). The peak for the combined non-pathogenic amoebae was among middle-aged subjects (age class 8, *n* = 3921 with a mean age of 34.8). The prevalence values for *E. histolytica/dispar* was too low to show any meaningful peak, but the highest prevalence was in age class 10 (mean age = 54.5, *n* = 2318).

### Sex of subjects

Prevalence values for male and female subjects are summarized in Table 2, where it can be seen that prevalence of each taxon was higher among the male subjects, in some cases almost twice as high as that among females (e.g. for *E. histolytica/dispar* x 1.8, and the lowest was for combined non-pathogenic amoebae at x 1.4). In all cases the difference in prevalence between the sexes was significant (for all four species combined, *χ*^2^_1_ = 56.8, *p* < 0.001; *B. hominis, χ*^2^_1_ = 20.7, *p* < 0.001; *G. duodenalis, χ*^2^_1_ = 10.5, *p* = 0.001; non-pathogenic amoebae, *χ*^2^_1_ = 6.5, *p* = 0.011; *E. histolytica/dispar, χ*^2^_1_ = 5.2, *p* = 0.022).

### Effect of interactions between factors affecting prevalence

**Fig. 3:**
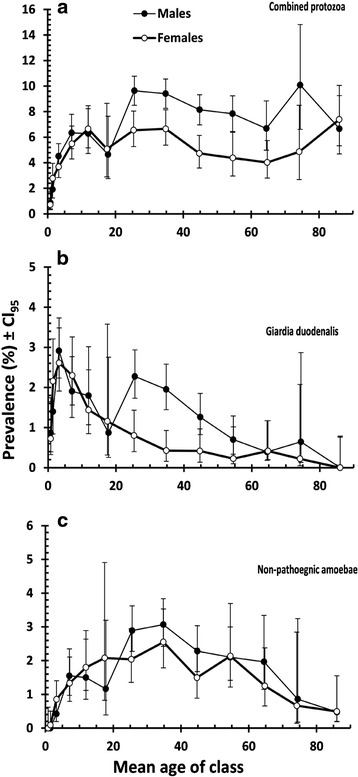
Age-prevalence profiles for male and females subjects. **a** combined protozoan infections; **b** *G. duodenalis* and **c** non-pathogenic amoebae. The sample sizes for male subjects in age classes 1–13 were *n* = 1040, 1360, 2129, 1419, 668, 344, 2594, 2509, 2061, 1428, 763, 466 and 210 respectively and for female subjects *n* = 827, 1113, 1762, 1350, 556, 433, 1374, 1412, 1203, 890, 720, 452 and 203 respectively

Significant interactions between SEX, AGE and INFECTION were found for all four species combined (*χ*^2^_12_ = 23.9, *p* = 0.021) and *G. duodenalis* (*χ*^2^_12_ = 22.2, *p* = 0.035) but not for *B. hominis,* non-pathogenic amoebae or *E. histolytica/dispar*. There was no difference in prevalence between the sexes among the young children. The discrepancy between the sexes became manifest in combined protozoa (Fig. 3a) from age class 6 (mean age = 17.6 ± 0.05) and in older age classes, but not in the very oldest age class (13, mean age = 86.1 ± 0.25). However, the pattern was quite different for *G. duodenalis*, which peaked in age class 3 (mean age = 3.3 ± 0.01) and then fell with increasing host age, notably among females but not to the same extent among males (Fig. 3b). In male subjects there was another resurgence of infection in age class 7 (mean age = 25.5 ± 0.04), and then a reduction thereafter among older subjects.

Figure 3c is included to show that there was no AGE x SEX x INFECTION interaction among the non-pathogenic amoebae, the age-prevalence curves for both males and females being very similar to one another right across all age classes. This interaction was not significant in any of the combinations tested when all the factors were included in models. Numerically the biggest discrepancies between the sexes were detected in the prevalences of combined protozoa (males = 5.1 %, CL_95_ = 4.040–6.399 and females = 2.63 %, CL_95_ = 1.818–3.669) and *B. hominis* (males = 3.92 %, CL_95_ = 2.986–5.060 and females = 1.54 %, CL_95_ = 0.943–2.385) among subjects from the Eastern Mediterranean. Similarly, the values for combined protozoan infections among Asian subjects were lower in females (males = 8.28 %, CL_95_ = 7.647–8.910 and females = 6.08 %, CL_95_ = 5.227–6.930) and also for *B. hominis* (males = 4.72 %, CL_95_ = 4.235–4.018 and females = 3.30 %, CL_95_ = 2.688–4.018) but in both cases, despite non-overlapping CL_95_ limits, with other factors taken into account, these differences between the sexes proved not to be significant.

### Combinations of protozoa

There were 27,548 uninfected subjects, 1522 subjects with just one taxon of protozoa, 190 with two, 25 with three and one with four taxa. Based on overall prevalence figures [15] predict that in the absence of interaction between species, and based only on prevalence values for each species in turn, it should be 27365; 18803; 40; zero and zero with no, one, two, three and four species respectively. This represents a significant difference to our data (*χ*^2^_4_ = 2668.1, *p* < 0.0001), implying that some protozoan infections were aggregated in particular subsets of the data. Consistent with this prediction, 27.4 % of the double taxa infections were identified in Africans and 53.2 % among Asians. Triple taxa infections were only encountered among Africans (36 %) and Asians (64 %) but the single case of four taxa was diagnosed in 2.8-year-old male Qatari citizen who harboured *G. duodenalis*, *C. mesnili*, *B. hominis* and *E. nana*.

## Discussion

In this paper, we have built on our earlier published studies and shown that after the peak in prevalence of intestinal protozoan infections among long-term residents and settled immigrants in Qatar which occurred in 2008 [11], the subsequent declining prevalence was largely sustained right through to 2014. A clear downward drift in the prevalence of intestinal protozoa was observed during the decade, from 7.98 % during Period 1 (2005–2008) [11], 5.13 % during Period 2 (2009–2011) [12] to 4.89 % during Period 3 (2012–2014). Falling temporal trends in prevalence of intestinal protozoan parasites have been observed elsewhere, as for example in Gaza where the prevalence of intestinal parasites has dropped significantly from 36.3 % in 1995 to 21.2 % in 2000 [16]. Likewise, over 6-year period among African refugees in Massechusetts in USA, prevalence was reported to have fallen by 10 %, from 57 to 47 % [17]. The overall pattern of temporal change in the prevalence of combined protozoan infections was clearly influenced by the corresponding pattern in the prevalence of *B. hominis*, the most common parasite recorded in the current study. *B. hominis* has been reported the most frequent species among immigrant workers, for example in Taiwan with prevalence of 3.4 % [18] and in Italy with prevalence of 52.7 % [19]. Whereas in most developing countries *E. histolytica/dispar* and/or *G. intestinalis* appear to be the dominant parasites, in developed countries *B. hominis* dominates as the most frequently recorded intestinal parasite [20]. The latter species is largely a misunderstood intestinal parasite capable of causing long-term infections in some individuals [21]. Our current data certainly underestimated the prevalence of *B. hominis* as higher prevalence can be detected by PCR (The prevalence was estimated to 71.1 % by PCR compared to 6.9 % by conventional microscopy [22]).

The second most prevalent protozoan parasites were the combined non-pathogenic amoebae. High prevalence of amoebae is usually found in subtropical and tropical regions with low hygienic standards and high population density [23] where waterborne outbreaks of amoebae are frequently observed [7]. The prevalence of non-pathogenic amoebae in the present study especially among individuals from Africa and Asia (2.58 and 1.96 % respectively), was higher than the pathogenic amoebae. Elsewhere, the prevalence of non-pathogenic amoebae has been reported not to exceed 20.0 % among migrants in Italy [19] and 9.2 % among Myanmar migrant workers in the Thai food industry [24].

The age-prevalence curve for *G. duodenalis* conforms to earlier reports of the parasite being mainly encountered among the youngest subjects of study group [e.g. 11]. Reduction in prevalence with increasing age is noted and is most likely associated with the development of immunity, as well as to altered patterns of behaviour (diet, water supply and improved hygienic measures) [25]. However, it is pertinent that we observed a resurgence of infection among young male subjects (mainly Asian) suggesting continued exposure throughout life. Those individuals are most likely to be the unskilled construction workers aggregating in overcrowded labour camps where conditions may be cramped and sanitary facilities limited and of a poor standard [13].

The higher prevalence of intestinal protozoan parasites among male compared with female subjects has been reported previously among immigrant workers. Our finding concurs with earlier observations concerning both protozoan [12] and helminth [13] infections among immigrants in Qatar. A similar pattern of protozoan infections has been observed in the USA among immigrants where male subjects were found to be more likely (OR 1.6) to carry the infections than females [17], as well as in Italy where the prevalence was 1.20 times higher in males than in females [21]. In some respects, a higher prevalence of parasitic infections among males is not surprising [26], explanations due to the difference in the behavioural and activities are far more likely to account for the male sex bias that we have observed.

In earlier work, we reported on the prevalence and diversity of helminth parasites in these same subjects. As expected, the prevalence of combined protozoan parasites was higher (5.93 % vs 1.86 %) [13]. Although prevalence of the intestinal protozoa in our study population fell with time, the reduction was not been as sharp and not to such low levels as that of the helminth infections in these same subjects [13]. The relatively high prevalence of intestinal protozoa and their longer persistence among immigrant communities in Qatar is then of particular interest. All the intestinal protozoa that we recorded are organisms whose infective stages are waterborne and can contaminate food and all are transmitted by the faecal-oral route. Their presence in the population is therefore indicative of inefficient sanitary systems, elevated environmental contamination, food and water contamination and poor personal hygienic behaviour by those affected. Our data suggest that some transmission does occur in Qatar as is evident by infections in Qatari subjects who are not immigrants. Indeed helminths have life cycles that are unlikely to be completed easily in the hot arid environment of Qatar, whereas protozoan infections may be transmitted more easily via contamination of water and food resources.

With its extended dry spells and drought, Qatar is highly dependent on importation of foods, which can include substandard, contaminated food items and result in the introduction of health hazards such as the transmission stages of intestinal protozoa as for example the observed contamination of shellfish and fresh products from Peru intended for export [27, 28]. Food contamination with parasites occurs during the production stage, from contaminated irrigation water, soil, untreated manure, or biosolids used as fertilizers [7]. Food may also become contaminated during the harvesting, handling, preparation processes, from cross-contamination with soiled implements, contaminated water used for preparation, or by the hands of the food handlers themselves [29, 30]. The risk of food-borne transmission is increased when food is consumed raw, undercooked, or in a semi-cooked form. Food-borne outbreaks associated with imported foods have been reported in several industrialized countries [31]. Concerning the waterborne transmission, all piped water in Qatar is supplied from desalination plants, and it is unlikely that these water supplies are the source of protozoan oocysts, cysts and trophozoites. However, if water is stored before consumption in overcrowded accommodation with poor sanitary facilities, local contamination is possible as the (oo)cysts of several protozoa are highly resistant to chlorination, a conventional water treatment method [20].

There is therefore a need for better awareness about these parasites and about strategies for improving hygiene habits, both in Qatar and in the countries of origin of the immigrant population. The transmission stages (oocysts/cysts) of some protozoa species can survive for a long time on uncleaned hands. Thorough, hand-washing is one of the most important interventions that has been proven to be extremely effective in curtailing the fecal–oral transmission of diseases [32].

## Conclusion

The declining prevalence of protozoan infections in Qatar indicates that improvements in public health have been made over the last decade and reflects the successful social integration of the immigrants who have come to work in the city. Nevertheless, there is still room for further improvements and in their overall strategy for improving the health of the inhabitants of Qatar, the public health authorities in Qatar should place a greater emphasis on how to reduce further intestinal protozoan infections. Improvements in regular inspections of the sanitary facilities in the labour work camps and hostels where the immigrant work force reside should be high on their list of priorities. Education and awareness program should be implemented also targeting both immigrants and residents about these infections and their modes of transmission. Personal hygiene practices should be emphasized and encouraged, as well mandatory food hazard control for the most susceptible sources of contamination.

## Abbreviations

CL_95_, 95 % confidence limits; HMC, Hamad Medical Corporation; OR, Odds ratio

## References

[1] Norman FF, Monge-Maillo B, Martínez-Pérez Á, Perez-Molina JA, López-Vélez R (2015). Parasitic infections in travelers and immigrants: part I protozoa. Future Microbiol.

[2] Tsiodras S (2016). Irregular migrants: a critical care or a public health emergency. Intensive Care Med.

[3] Jad B, Dogra S, Mahajan B (2015). Significant decrease in prevalence of intestinal parasites among patients seeking treatment in a tertiary care hospital in Jammu: a changing trend. Int J Curr Microbiol App Sci.

[4] Poulakou G, Bassetti M, Timsit JF (2016). Critically ill migrants with infection: diagnostic considerations for intensive care physicians in Europe. Intensive Care Med.

[5] Mezeid N, Shaldoum F, Al-Hindi AI, Mohamed FS, Darwish ZE (2014). Prevalence of intestinal parasites among the population of the Gaza Strip, Palestine. Ann Parasitol.

[6] Leung PO, Chen KH, Chen KL, Tsai YT, Liu SY, Chen KT (2014). Epidemiological features of intestinal infection with *Entamoeba histolytica* in Taiwan, 2002–2010. Travel Med Infect Dis.

[7] World Health Organization (WHO). Infectious diseases of potential risk for travellers. 2009. Available from: http://www.who.int/ith/ITH2009Chapter5.pdf.

[8] Molyneux DH (2004). “Neglected” diseases but unrecognized successes – challenges and opportunities for infectious disease control. Lancet.

[9] Pierce KK, Kirkpatrick BD (2009). Update on human infections caused by intestinal protozoa. Curr Opin Gastroenterol.

[10] Abu-Madi MA, Behnke JM, Ismail A (2008). Patterns of infection with intestinal parasites in Qatar among food handlers and housemaids from different geographical regions or origin. Acta Trop.

[11] Abu-Madi MA, Behnke JM, Doiphode SH (2010). Changing trends in intestinal parasitic infections among long-term-residents and settled immigrants in Qatar. Parasit Vectors.

[12] Abu-Madi MA, Behnke JM, Doiphode SH (2013). Intestinal parasitic infections among long-term-residents and settled immigrants in Qatar in the period 2005 to 2011. Am J Trop Med Hyg.

[13] Abu-Madi MA, Behnke JM, Boughattas S, Al-Thani A, Doiphode SH, Deshmukh A (2016). Helminth infections among long-term-residents and settled immigrants in Qatar in the decade from 2005 to 2014: temporal trends and varying prevalence among subjects from different regional origins. Parasit Vectors.

[14] Rohlf FJ, Sokal RR (1995). Statistical Tables.

[15] Janovy J, Clopton RE, Clopton DA, Snyder SD, Efting A, Krebs L (1995). Species density distributions as null models for ecologically significant interactions of parasite species in an assemblage. Ecol Modell.

[16] El Kichaoi AY, Abd Rabou AFN, Sharif FA, Husni M, El-Amssi HM (2004). Changing trends in frequency of intestinal parasites in Gaza, 1995–2000. J Islamic Univ Gaza, (Natural Sciences Series).

[17] Garg PK, Perry S, Dorn M, Hardcastle L, Parsonnet J (2005). Risk of intestinal helminth and protozoan infection in a refugee population. Am J Trop Med Hyg.

[18] Wang LC (2004). Changing patterns in intestinal parasitic infections among Southeast Asian laborers in Taiwan. Parasitol Res.

[19] Gualdieri L, Rinaldi L, Petrullo L, Morgoglione ME, Maurelli MP, Musella V, Piemonte M, Caravano L, Coppola MG, Cringoli G (2011). Intestinal parasites in immigrants in the city of Naples (southern Italy). Acta Trop.

[20] Fletcher SM, Stark D, Harkness J, Ellis J (2012). Enteric protozoa in the developed world: a public health perspective. Clin Microbiol Rev.

[21] Calderaro A, Montecchini S, Rossi S, Gorrini C, De Conto F, Medici MC, Chezzi C, Arcangeletti MC (2014). Intestinal parasitoses in a tertiary-care hospital located in a non-endemic setting during 2006–2010. BMC Infec Dis.

[22] Abu-Madi M, Aly M, Behnke JM, Clark CG, Balkhy H (2015). The distribution of *Blastocystis* subtypes in isolates from Qatar. Parasit Vectors.

[23] Herbinger KH, Fleischmann E, Weber C, Perona P, Loscher T, Bretzel G (2011). Epidemiological, clinical, and diagnostic data on intestinal infections with *Entamoeba histolytica* and *Entamoeba dispar* among returning travelers. Infection.

[24] Nuchprayoon S, Sanprasert V, Kaewzaithim S, Saksirisampant W (2009). Screening for intestinal parasitic infections among Myanmar migrant workers in Thai food industry: A high-risk transmission. J Immigr Minor Health.

[25] Heimer J, Staudacher O, Steiner F, Kayonga Y, Havugimana JM, Musemakweri A, Harms G, Gahutu JB, Mockenhaupt FP (2015). Age-dependent decline and association with stunting of *Giardia duodenalis* infection among schoolchildren in rural Huye district. Rwanda Acta Trop.

[26] Klein SL (2004). Hormonal and immunological mechanisms mediating sex differences in parasite infection. Parasite Immunol.

[27] Nichols GL (1999). Food-borne protozoa. Brit Med Bull.

[28] USDA. Protozoan contaminants in shellfish. 2003; http://fsrio.nal.usda.gov/nal_web/fsrio/printresults.php?ID=1635

[29] Motazedian MH, Najjari M, Ebrahimipour M, Asgari Q, Mojtabavi S, Mansouri M (2015). Prevalence of intestinal parasites among food-handlers in Shiraz, Iran. Iran J Parasitol.

[30] WHO. The present state of foodborne disease in OECD countries. 2003; http://www.who.int/foodsafety/publications/foodborne_disease/oecd_fbd.pdf

[31] Ho AY, Lopez AS, Eberhart MG, Levenson R, Finkel BS, da Silva AJ, Roberts JM, Orlandi PA, Johnson CC, Herwaldt BL (2002). Outbreak of Cyclosporiasis associated with imported Raspberries, Philadelphia, Pennsylvania, 2000. Emerg Infect Dis.

[32] Alum A, Rubino JR, Ijaz MK (2010). The global war against intestinal parasites-should we use a holistic approach?. Int J Infect Dis.

